# *FASTER MT*: Isolation of Pure Populations of *a* and α Ascospores from *Saccharomyces* *cerevisiae*

**DOI:** 10.1534/g3.111.001826

**Published:** 2012-04-01

**Authors:** Brian L. Chin, Margaret A. Frizzell, William E. Timberlake, Gerald R. Fink

**Affiliations:** Whitehead Institute for Biomedical Research, Cambridge, Massachusetts 02142

**Keywords:** budding yeast, red fluorescent protein, *MATa*, fluorescence-activated cell sorting, hygromycin resistance, *BUD5-TAF2*

## Abstract

The budding yeast *Saccharomyces cerevisiae* has many traits that make it useful for studies of quantitative inheritance. Genome-wide association studies and bulk segregant analyses often serve as first steps toward the identification of quantitative trait loci. These approaches benefit from having large numbers of ascospores pooled by mating type without contamination by vegetative cells. To this end, we inserted a gene encoding red fluorescent protein into the *MATa* locus. Red fluorescent protein expression caused *MATa* and *a*/α diploid vegetative cells and *MATa* ascospores to fluoresce; *MAT*α cells without the gene did not fluoresce. Heterozygous diploids segregated fluorescent and nonfluorescent ascospores 2:2 in tetrads and bulk populations. The two populations of spores were separable by fluorescence-activated cell sorting with little cross contamination or contamination with diploid vegetative cells. This approach, which we call *F*luorescent *As*cospore *T*echnique for *E*fficient *R*ecovery of *M*ating *T*ype (*FASTER MT*), should be applicable to laboratory, industrial, and undomesticated, strains.

Mapping and identification of quantitative trait loci (QTL) are the keys to understanding complex traits in humans, animals, plants, and eukaryotic microorganisms (Lander and Schork 1994; Darvasi 1998; Gianni *et al.* 2009; Jing *et al.* 2010; Jimenez-Gomez *et al.* 2011). Such studies often use hundreds or even thousands of individuals (Churchill and Doerge 1994) to detect associations or linkages between genetic markers, such as single nucleotide polymorphisms, and traits of interest. *Saccharomyces cerevisiae* (yeast) is well suited for QTL mapping studies. Its facile genetic system, small genome size, and lack of extensive repeated DNA make it ideal for developing strategies to detect the many loci contributing to complex traits in eukaryotes. Furthermore, precise control of the cellular environment when growing yeast minimizes nongenetic variability and thereby increases the ability to detect quantitative variation caused by genetic differences. The potential for yeast to help solve basic problems in quantitative genetics has been, for example, exploited in studies of sporulation (Deutschbauer and Davis 2005), heat tolerance (Steinmetz *et al.* 2002), and chemical tolerance (Ehrenreich *et al.* 2010).

In yeast, meiotic segregants can be isolated by micromanipulation of individual tetrads to separate the four ascospores or as random spores, where ascus walls are enzymatically removed and the population of released spores is plated. Because tetrad analysis is time consuming and not automated it is ill suited to produce sufficient numbers of recombinant progeny for QTL studies. Isolation of large numbers of random spores without micromanipulation is straightforward but has at least two technical shortcomings. First, a diploid culture subjected to meiosis-inducing conditions contains contaminating diploids that failed to undergo meiosis in addition to the desired haploid meiotic spores. Second, the population of haploid meiotic cells consists of equal numbers of the two mating types, which when plated could mate to form diploids. Without a method for removing diploids and separating haploids into *a* and α mating types, the random spore population is not useful for QTL mapping. Thus, simple, rapid, and efficient methods for bulk isolation of pure ascospores sorted by mating type are needed.

Rapid separation of haploids and diploids has been accomplished by incorporation of genetic markers that allow for selection by (1) insertion of a gene-promoter construct expressed only in haploids of one mating type and (2) the use of a recessive resistance marker [*e.g.*, canavanine resistance (Whelan *et al.* 1979)] to select against diploids (Tong and Boone 2007; Ehrenreich *et al.* 2010). Although effective, these approaches require the introduction of engineered cassettes via multiple manipulations and entail selections that could bias some analyses. Further, they may not be applicable to wild strains, which are rich sources of quantitative variation but are diploid, often homothallic, and lack genetic markers needed for introduction of some engineered cassettes (Timberlake *et al.* 2011).

Thacker *et al.* 2011 demonstrated the feasibility of obtaining ascospore-autonomous expression of fluorescent protein constructs and used these to visualize meiotic events. Fluorescently tagged ascospores would be well suited for preparation of QTL mapping populations if expression of the tag could be limited to one mating type. The approach we describe here is based on the integration of a red fluorescent protein (RFP) gene at the *MATa* locus, with selection provided by a hygromycin-resistance gene so that the cassette can be introduced into any transformable, haploid or diploid, hygromycin-sensitive strain. *MATa* vegetative cells and ascospores thus tagged contain a visible marker useful for separation of cells by hand or fluorescence-activated cell sorting (FACS).

## Materials and Methods

We used standard yeast molecular genetic techniques (Guthrie and Fink 2004; Amberg *et al.* 2005) to obtain the *S. cerevisiae* ∑1278b (http://wiki.yeastgenome.org/index.php/History_of_Sigma) strains given in Table 1.

**Table 1 S.** *cerevisiae* strains used in the study

Strain	Genotype
ML1	*ura3-52/ura3-52 his3*::*hisG/HIS3 leu2*::*hisG/LEU2 trp1*::*hisG/TRP1 tec1*::*KANMX/TEC1 MATa (mata2*::*yEmRFP–HYGMX)/MAT*α
ML2	*ura3-52/URA3 his3*::*hisG/his3*::*hisG leu2*::*hisG/LEU2 trp1*::*hisG/trp1*::*hisG tec1*::*KANMX/TEC1 MATa/MAT*α
ML3	*ura3-52 leu2*::*hisG MATa (mata2*::*yEmRFP-HYGMX)*
ML4	*ura3-52 leu2*::*hisG MAT*α
ML5	*his3*::*hisG trp1*::*hisG tec1*::*KANMX MATa*
ML6	*his3*::*hisG trp1*::*hisG tec1*::*KANMX MAT*α

**Figure 1:**
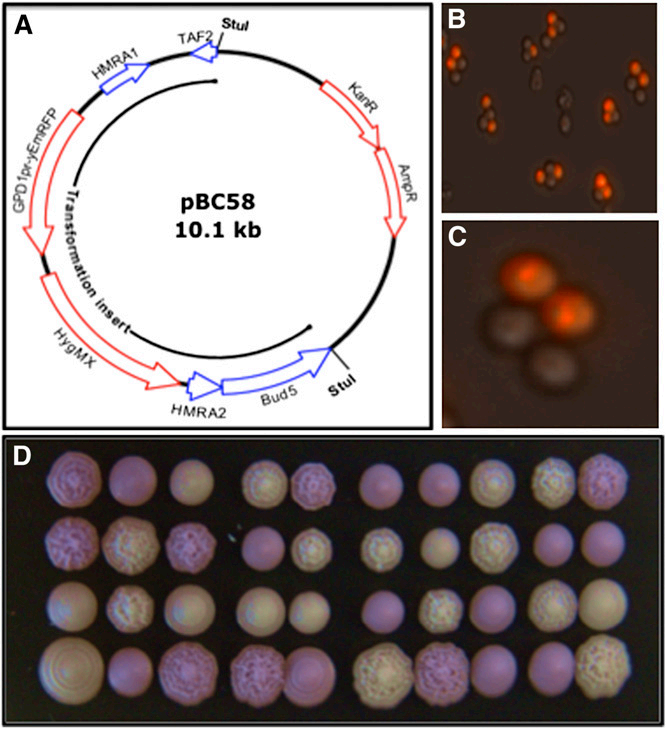
Transformation with the RFP Cassette. (A) Plasmid pBC58. The RFP-hyg^R^ cassette was inserted between the first and second codons of *HMRa2*. The figure retains the *HMRa* notation because the *MATa* sequence was first inferred from the sequence of the silenced locus. However, the cassette’s homology extends to the flanking *TAF2* and *BUD5* genes so transformation with the *Stu*I fragment is directed to the *MAT* locus. (B) and (C) Fluorescence phenotype of asci. Most of the intact asci we observed contained two fluorescent and two nonfluorescent spores. The RFP appeared to accumulate in vacuoles. (D) Growth of tetrads. Dissected tetrads were grown at 30° on YPD medium, incubated at 4° for several days to enhance fluorescence, and photographed under ambient light. Normal segregation of fluorescent ascospores shown in (B) and (C) was replicated in these and all other tetrads we observed. We confirmed that *a* mating type, fluorescence, and hygromycin resistance were completely linked. By contrast, the variations in colony morphology shown in the figure were unlinked to fluorescence.

Plasmid pBC58 (Figure 1A) was constructed as follows: A *Bam*H1 fragment from plasmid yEpGAP-Cherry (Keppler-Ross *et al.* 2008) containing a yeast-optimized red fluorescent protein gene and promoter (*TDH3p_yEmRFP_*) was cloned into pAG35 (Goldstein and McCusker 1999). A polymerase chain reaction (PCR) product (BCP538-539; Table 2) encompassing the RFP-hygMX genes and adding approximately 50 bp of homology at the 5′ end of *MATa2* was used to direct integration at *MATa*. A second PCR product (BCP569-571; Table 2) spanning *MATa* and adding terminal *Stu*I sites was then made from genomic DNA and cloned into pCR TOPO2.1 (Invitrogen) to produce pBC58, which is available upon request.

**Table 2:** Primers used in the study

Primer	Sequence
BCP538	5′-TGCAAACAACATCTCAACTCACTACTACCATTACTGTATT ACTCAAAGAAGAAGCTTCGTACGCTGCA
BCP539	5′-TTTTTCTGTGTAAGTTGATAATTACTTCTATCGTTTTCT ATGCTGCGCATATCGATGAATTCGAGCTCG
BCP569	5′-AGGCCTGTTAGAAAAGTGGAAAAACAAAT
BCP571	5′-AGGCCTTATCAGTTAGACCAATGTAATGAA

Cells were examined with a 40×/0.75 M/N2 dry objective or 100×/1.30 H/N2 oil immersion objective at room temperature. Fluorescence was monitored at 590 nm with a G-2E/C blocking filter (Nikon). FACS was performed with either a BD Biosciences FACS AriaIIU SORP or LSRII SORP with the 561 nm laser and 610/20 filter.

Growth curves were performed in microtiter plates with 150 µL of medium/well. Wells were inoculated with 10 µL of 1 OD_600_/mL aqueous suspensions of cells. Plates were incubated at 30° and OD_600_ measurements were taken at 30-min intervals after shaking for 15 sec.

Ascospores were isolated by scraping well-sporulated colonies from SM plates, suspending them in 1 mL of phosphate-buffered saline, and adding 1000 units of lyticase (Sigma-Aldrich). After incubation at 30° for 8 hr, sodium dodecyl sulfate was added to 1%. The ascospores were washed twice with 0.1% Tween-20, 5 mM EDTA, and suspended at approximately 10^9^/mL.

## Results and Discussion

Transformation of haploid *MATa* strains with the *Stu*I fragment of pBC58 (Figure 1A) resulted in the formation of hygromycin-resistant (hyg^R^), pink colonies. The intensity of the color increased upon incubation at 4°. We crossed one transformant to produce heterozygous diploid ML1 (Table 1), whose color was approximately one-half as intense as that of the haploid. Figure 1, B−D shows that segregation of the marker in ML1 tetrads was 2 RFP^+^:2 rfp^−^. PCR analysis of transformants indicated that a single copy of RFP-hygMX had integrated at *MATa*. Moreover, mating type was completely linked to RFP in 20 tetrads. These results indicate that transformation was due to integration by homology at *MATa*.

Transformation of a diploid strain with the *Stu*I fragment also resulted in formation of hyg^R^, pink colonies. Of these, approximately 10% were converted from *a*/α to *a*/*a* diploids, as evidenced by acquisition of mating competence with a *MATα* tester lawn. This is predicted by transplacement of the *MAT*α locus by the pBC58 *Stu*I fragment, which contains homologous sequences flanking *MAT* (*BUD5/TAF2*; Figure 1A). The ability to make RFP^+^/rfp^−^ diploids by transformation speeds up analysis because strains can be sporulated without intermediate steps to obtain segregants. Further, Klar (1980) showed that *a*/*a* diploids could be induced to sporulate after transient mating with a *MAT*α haploid containing a *kar1* mutation that interfered with karyogamy (Conde and Fink 1976). This approach, which is expected to produce equal numbers of *MATa* spores containing and lacking the *RASTER* insert, could be used to obtain untagged *MATa* populations.

**Figure 2:**
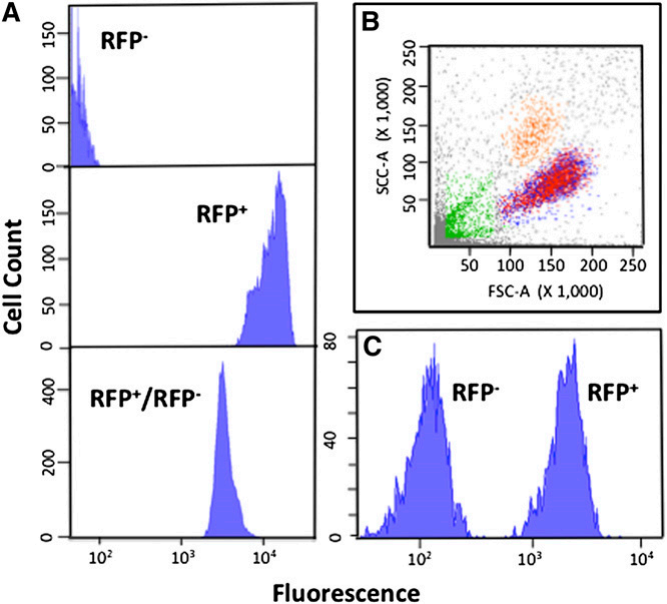
FACS. (A) Vegetative cells. Top: nontransformed *MATα* haploids; middle: transformed *MATa* haploids; bottom: heterozygous diploid. (B) Separation of ascospores. An ascospore suspension was subjected to FACS. We determined that the population of cells centered at approximately 150 FSC-A and approximately 90 SSC-A (×1000) contained single cells, whereas the other populations contained either aggregates or debris. (C) Separation of fluorescent and nonfluorescent ascospores. The target population from (B) was further separated into cells with low and high fluorescence (characterized in Table 3).

We subjected vegetative cells and ascospores to FACS to assess the feasibility of separating them by mating type. Figure 2A shows that control haploid cells (nontransformed or *MAT*α derivatives of transformed diploids) and transformed haploids are separated by approximately 3 logs of intensity, whereas heterozygous diploids are intermediate. Gating permitted separation of the three classes: diploids and *MATa* and MATα haploids. Separation of ascospores is more relevant to most studies. Figure 2B shows that forward and side scatter analysis separated a crude ascospore preparation into four populations, one of which contained equal numbers of individual fluorescent and nonfluorescent cells. Microscopic examination of these cells showed that they were unaggregated ascospores. Figure 1C shows that this population could be sorted into nonoverlapping, nonfluorescent and fluorescent subpopulations, present in equal proportions.

We tested each population for viability and cross-contamination (*MATa*→*MAT*α and converse). Table 3 shows that spore viability was high (60%–70%) even after the rigorous enzymatic and detergent treatments used to eliminate ascus walls and vegetative cells, and FACS. For the *MATα* (nonfluorescent) population, the contamination with hyg^R^ cells was <0.2%, which should be acceptable for most purposes. Moreover, as the contaminating cells, which we presume are the result of aggregation, are RFP^+^, they can be removed without much effort after plating because the colonies are red. The *MATa* (fluorescent) population was contaminated with approximately 0.2% of fluorescent diploid cells (Table 3). These could be removed by further enzymatic and detergent treatments.

**Table 3:** Characteristics of sorted ascospores

Parameter Tested	Sorted Ascospores	
	RFP^−^	RFP^+^
Physical count*^a^*	6.7 × 10^6^	6.3 × 10^6^
Viable count*^b^*	4.2 × 10^6^	4.5 × 10^6^
Viability, %	63	71
Contamination with Hyg^R^ cells, %*^c^*	0.16	N/A
Contamination with diploid cells, %*^d^*	N/A	0.21

a Counted in a hemocytometer.

b Serial dilutions were spread onto YPD plates and colonies were counted after 2 days at 30°.

c RFP-negative cells (2 × 10^3^ CFU/plate) were spread onto YPD plates containing 200 µg/mL of hygromycin-B. Colonies were counted after 3 days at 30°.

d RFP-positive cells (20−50 CFU/plate) were grown on YPD for 2 days at 30° and replica-plated onto lawns of a *MATα* tester strain. After 2 days at 30°, the colonies were scored for halo formation. Diploids were implicated by lack of halo formation.

These results lead to the following conclusions:

1. Large, pure populations of *MATa* and *MAT*α spores can be obtained by FACS. These have high viability making them suitable for GWAS and BSA.
2. RFP is expressed at high enough levels to be detected visually in colonies. Therefore, because RFP and hyg^R^ are completely linked to *MATa*, haploid colonies can be separated into mating types by fluorescence or drug resistance.
3. The ability to use both *MATa* and *MAT*α populations lacking the introduced marker provides a way to get around potential distortions arising from linkage of genes of interest to *MAT*.

**Figure 3:**
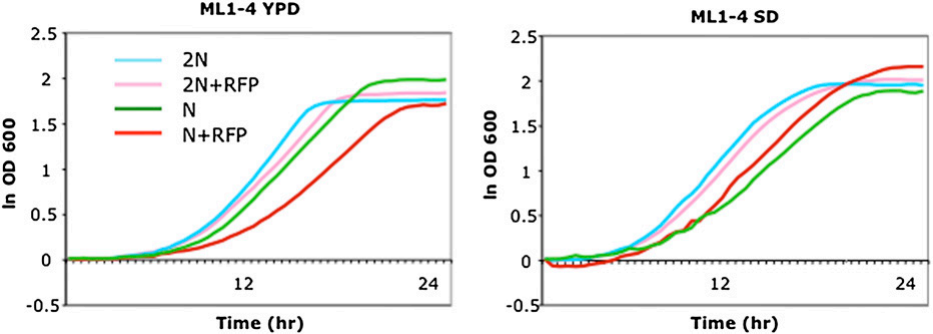
Growth characteristics of RFP^+^ and RFP^−^ strains. Strains ML1-4 (Table 1) were grown in a microtiter plate and the OD_600_ was recorded every 0.5 hr. ODs were converted to natural logs, and the zero-time values were subtracted from each time point. YPD, yeast extract, peptone, glucose medium; SD, synthetic glucose medium supplemented for the requirements of the strains used (Amberg *et al.* 2005).

Although the deletion of *MATa2* has been reported to have no effect on growth, mating, or sporulation (Dranginis 1989), we assessed the growth characteristics and mating competence of some of our *MATa2* transplacement strains. Figure 3 shows growth curves of strains ML1-4 (Table 1). ML1, a diploid containing the RFP cassette, and ML2, a related diploid lacking the cassette, had similar growth profiles on either YPD or supplemented SD, although ML1 reproducibly grew a little slower. By contrast, ML3, a haploid containing the cassette, grew much more slowly and to a lower final OD in YPD than isogenic ML4 lacking the cassette. However, this difference was moderated and reversed in SD. These differences could be a consequence of the insertion of two strong promoters at *MATa2* and suggest that controlled measurements of growth rates (or other traits of interest) are required for strains containing the RFP cassette. Of course this caution applies to any strains carrying residual markers, selection cassettes, chromosome abnormalities, etc., introduced to facilitate QTL studies, because they might modify or bias traits of interest directly or indirectly.

We found that RFP strains mated as well as non-RFP strains in routine strain constructions. However, in a mating assay where congenic RFP^+^ and rfp^−^ strains were in competition for a common mating partner the RFP^+^ strain mated somewhat less efficiently than the rfp^−^ strain. This disadvantage decreased with increased mating time. Dranginis, 1989, reported that strains containing a complete deletion of *MATa2* had normal mating characteristics, but this conclusion was not based on the sensitive competitive assays employed here. Whatever the function of *MATa2* and the effect of the insertion, RFP strains in which it is disrupted mate well under the standard, noncompetitive conditions used for strain construction.

These results lead to the following conclusions:

1. Integration of the RFP cassette at *MATa* does not influence growth rate on one medium but does on another. Growth rates of selective markers should be assessed in QTL studies.
2. The RFP cassette does not interfere with standard genetic manipulations, but may reduce mating efficiency in more sensitive assays.

### Summary

Integration of a cassette containing RFP and hyg^R^ into the *MATa* locus provides a simple, robust means for marking mating type so that *a* and α ascospores can be separated and purified by FACS. The fact that the cassette can be transformed into most haploid or diploid *S. cerevisiae* strains without introduction of other mutations means that it should be useful for studies of quantitative inheritance in laboratory, industrial, and wild strains. Moreover, it can serve as a mating type indicator without compromising other genotypic or phenotypic features.
